# Aspartate aminotransferase/alanine aminotransferase ratio and subsequent cancer development

**DOI:** 10.1002/cam4.4473

**Published:** 2021-12-01

**Authors:** Daiki Kobayashi, Kazuki Yamamoto, Takeshi Kimura, Takuro Shimbo

**Affiliations:** ^1^ Division of General Internal Medicine Department of Medicine St. Luke’s International Hospital Tokyo Japan; ^2^ Department of Epidemiology St. Luke's International University Graduate School of Public Health Tokyo Japan; ^3^ Fujita Health University Toyoake Japan; ^4^ Department of General Medicine Juntendo University Faculty of Medicine Tokyo Japan; ^5^ Department of Gastroenterology St. Luke’s International Hospital Tokyo Japan; ^6^ The Center for Preventive Medicine St. Luke’s International Hospital Tokyo Japan; ^7^ Ohta Nishinouchi Hospital Koriyama Japan

**Keywords:** alanine aminotransferase, aspartate aminotransferase, AST/ALT ratio, Japan, malignancy

## Abstract

**Background:**

We aimed to evaluate the association between the aspartate aminotransferase (AST)/alanine aminotransferase (ALT) ratio and subsequent development of any type of cancer in an apparently healthy population.

**Methods:**

We conducted a retrospective cohort study at St. Luke's International Hospital, Tokyo, Japan between 2005 and 2018. All participants who visited for voluntary health checkups were included. We divided the participants into different quintiles based on the baseline AST/ALT ratios and examined the outcomes.

**Results:**

A total of 85,658 participants were included. The mean age was 44.7 years (standard deviation 12.0) at baseline, and 42,913 (50.1%) of them were men. During a median follow‐up of 61.6 months, 4701 (5.5%) participants developed some type of cancer. Compared with the middle AST/ALT ratio group, no other groups had similar adjusted hazard ratios (HR) for the development of any type of cancer in both men and women. When stratified by alcohol consumption, very high (adjusted HR 1.36; 95% CI 1.13–1.63) and high (adjusted HR 1.26; 95% CI 1.05–1.50) AST/ALT ratio groups among men who were regular drinkers had increased adjusted HRs for any type of cancer development, but the very high AST/ALT ratio group among men who were abstainers (adjusted HR 0.64; 95% CI 0.42–0.97) and very low AST/ALT ratio group among men who were occasional drinkers (adjusted HR 0.69; 95% CI 0.48–0.98) had lower adjusted HRs compared with the middle AST/ALT ratio group. Among women, regardless of alcohol consumption, adjusted HR for any type of cancer development was similar across all AST/ALT ratio groups.

**Conclusion:**

People with higher AST/ALT ratios tended to have a higher risk of developing any type of cancer among men who were regular drinkers, but this risk was lower among men who were abstainers. Among women, regardless of alcohol consumption, there was no association between the development of any type of cancer and AST/ALT ratio.

## Graphical abstract

People who had higher aspartate aminotransferase (AST)/alanine aminotransferase (ALT) ratio tend to have a higher risk of developing any type of cancer compared with those with middle AST/ALT ratio, especially regular drinkers. Cancer development in the digestive organ, other than hepatocellular carcinoma; urinary tract; or respiratory and intrathoracic organs may be associated with AST/ALT ratio.

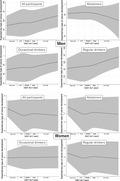

## 1 INTRODUCTION

Liver enzymes, such as aspartate aminotransferase (AST) and alanine aminotransferase (ALT), are associated with not only the development and prognosis of hepatocellular carcinoma and liver metastasis but also other types of cancer. In patients infected with the hepatitis C virus, elevated ALT is considered to be an independent risk factor for hepatocellular carcinoma.^1^ A previous study reported that elevated liver function tests were positively correlated with gallbladder cancer.^2^ Elevated serum liver enzymes, such as alkaline phosphatase, total and direct bilirubin, and γ‐glutamyl transpeptidase, were associated with poor prognosis in patients with intrahepatic cholangiocarcinoma.^3^ In terms of cancer types occurring in organs other than the hepatobiliary tract, liver function tests were associated with all‐cause mortality among patients with cancer.^4^, ^5^ Like intrahepatic cholangiocarcinoma, ALT was reported to be an independent prognostic factor of extrahepatic cholangiocarcinoma.^6^ Hence, liver enzymes may be associated with the evaluation or prognosis of several types of cancer.

Among liver enzymes, AST/ALT ratio is considered for the prediction of prognosis in patients with certain cancers. A low AST/ALT ratio was an independent prognostic factor of long‐term survival among patients with primary hepatic carcinoma.^5^ In terms of cancer types other than those affecting the hepatobiliary tract, an elevated serum AST/ALT ratio was associated with better prognosis in patients with gastric,^7^ oral and oropharyngeal,^8^ pancreatic,^9^ and prostate cancers.^10^ Therefore, the AST/ALT ratio would be useful for predicting survival in patients with cancer.

Currently, it is speculated that the AST/ALT ratio may be used to diagnose certain cancers, although only a limited number of studies have been published. An AST/ALT ratio >1 may suggest the existence of primary hepatic cancer among patients with positive hepatitis B surface antigens.^11^ Another study reported that the AST/ALT ratio is an independent predictive factor of prostate cancer among patients with benign prostatic hyperplasia.^10^

Although several studies have suggested that an AST/ALT ratio can be used as a prognostic or diagnostic factor in patients with cancer, limited studies have evaluated the association between the AST/ALT ratio and future cancer development among patients without cancer. We hypothesized the biological mechanisms underlying the association between the AST/ALT ratio and subsequent cancer development. Low ALT levels are said to be associated with free radicals and oxidative stress^12^ causing cancer, whereas increased AST levels are said to be related to higher cancer proliferation rates and more severe tissue damage.^8^ As a result, decreased ALT and increased AST levels, causing a high AST/ALT ratio, may be related to increased risk. Therefore, we aimed to evaluate the association between AST/ALT ratio and subsequent development of any type of cancer.

## 2 METHODS

We conducted a retrospective cohort study at St. Luke's International Hospital, Tokyo, Japan, between 2005 and 2018. All participants who visited the Center for Preventive Medicine at St. Luke's International Hospital for voluntary health checkups were included, and their data were obtained from St. Luke's Health Checkup Database. Patients with histories of cancer at baseline (3315) were excluded. The primary outcome was the development of any type of cancer. As a subanalysis, development of each type of cancer was evaluated during the follow‐up. We divided the participants based on the baseline AST/ALT ratios and examined their outcomes by groups; and, the third quintile group was considered as the reference group. St. Luke's Ethics Committee Institutional Review Board approved this study (approval number: 18‐R203, comprehensive approvals for the studies on environmental risk factors or clinical information).

### 2.1 Cancer development

The primary outcome was the development of any type of cancer during the follow‐up. As a subanalysis, the development of each type of cancer based on the International Classification of Diseases, tenth revision, and major organ‐specific cancers were evaluated. The data on cancer development was obtained from the participants’ self‐reports, their electronic medical records, or the hospital's cancer registry. The participants were asked about their current/past medical history, including the development of cancer, as a part of the health checkups each time they visited the center. All the treatments or examinations performed in the hospital were recorded in their electronic medical records. Finally, the hospital's cancer registry included patients’ information collected by trained staff from the referral documents from other hospitals or via interviews with the patients or their family members.

### 2.2 AST, ALT, and AST/ALT ratio

As a part of the heath checkups, the absolute values of serum AST (IU/L) and ALT (IU/L) levels of all the participants were measured. We used AST, ALT, and their ratio (AST/ALT) measured at baseline as exposure variables in this study. Further, we divided the participants into different quintiles based on the AST/ALT ratio and compared their outcomes, with the third quintile group assigned as the reference group, because there was no appropriate clinical category for the AST/ALT ratio to the best of our knowledge.

### 2.3 Potential confounders

Regarding potential confounders, data on the participants’ demographic characteristics, environmental risk factors, and medical histories were obtained using questionnaires administered during the health checkup. The demographic data included the participants’ age and sex. Environmental risk factors included smoking status (never, former, or current smoker), alcohol consumption status (abstainer, occasional drinker, or regular drinker), and exercise habits (almost none, 1–2 times a week, 3–5 times a week, and almost every day). This information was self‐reported from the response to the questionnaire and no further detailed information was available. Data on the current history of hypertension or diabetes and family history of any type of cancer at baseline were collected from the participants’ reports. Fatty liver was also examined based on the results of the ultrasound conducted as a part of the baseline health checkup. In addition, the body mass indices were calculated based on the participants’ heights and weights measured by trained staff and were categorized as follows based on the World Health Organization's Asian criteria: underweight (<18.5 kg/m^2^), normal weight (>18.5 to ≤24.9 kg/m^2^), and overweight/obesity (>24.9 kg/m^2^).

### 2.4 Statistical methods

First, we compared the participants’ baseline characteristics based on the AST/ALT ratios. Then, the Kaplan–Meier curves for any type of cancer‐free survival were drawn based on the AST/ALT ratio category and a univariate analysis using a log‐rank test was performed. In the multivariable analyses, we used the Cox proportional hazard model to compare the outcomes of the different AST/ALT ratio categories, while adjusting for potential confounders. The test of proportional‐hazards assumption using Schoenfeld residual was applied. Because lifestyles and cancer development would vary between men and women, we performed the main analyses separately by sex. As the AST/ALT ratio may be affected by the alcohol consumption status, a subgroup analysis stratified by alcohol consumption status was performed. Effect modification of sex or alcohol consumption status on the association between cancer development and the AST/ALT ratio was examined to justify the stratification. We also conducted restricted cubic spline analyses with knots at quintile AST/ALT ratio categories.^13^ In addition, sensitivity analyses were performed excluding high AST and ALT levels (>50, >60, and >70 IU/L for both AST and ALT), as these levels may reflect an underlying undiagnosed disease that may contribute to the development of cancer. Moreover, another sensitivity analysis excluding participants with fatty liver was performed, because fatty liver, including non‐alcoholic steatohepatitis, is known to be an independent risk factor for certain cancers.

All the analyses were performed using Stata MP 14.2 in 2021 (STATA Corp.).

## 3 RESULTS

A total of 85,658 participants were included in this study. The participants’ mean age was 44.7 years (standard deviation [SD]: 12.0), and 42,913 (50.1%) of the participants were men. The mean AST level was 21.7 IU/L (SD 10.0), whereas the mean ALT level was 22.5 IU/L (SD 16.7). The participants were divided into different quintiles according to the AST/ALT ratios (first quintile [very low]: <0.81, second quintile [low]: >0.81 to <1.00, third quintile [middle]: >1.00 to <1.19, fourth quintile [high]: >1.19 to <1.40, and fifth quintile [very high]: >1.40). Those with higher AST/ALT ratios tended to be women, abstainers, and never smokers, but they had poor exercise habits (Table 1). In addition, they were more likely to have normal weights and less likely to have fatty liver, diabetes, and hypertension.

**TABLE 1:** Baseline participant characteristics by AST/ALT ratio category

	AST/ALT ratio					Total (*n* = 85,655)
	First quintile <0.81 (*n* = 17,229)	Second quintile >0.81, <1.00 (*n* = 18,177)	Third quintile >1.00, <1.19 (*n* = 16,087)	Fourth quintile >1.19, <1.40 (*n* = 17,363)	Fifth quintile >1.40 (*n* = 16,799)	
Age, years, mean (SD)	43.8 (10.4)	46.1 (11.8)	45.9 (12.3)	44.7 (12.5)	42.9 (12.8)	44.7 (12.0)
Men, *n* (%)	14,710 (85.4)	11,800 (64.9)	7400 (46.0)	5431 (31.3)	3571 (21.3)	42,912 (50.1)
Alcohol consumption, *n* (%)						
Abstainer	5854 (34.0)	6473 (35.6)	6387 (39.7)	7314 (42.1)	7145 (42.5)	33,173 (38.7)
Occasional	3278 (19.0)	3033 (16.7)	2769 (17.2)	3018 (17.4)	2825 (16.8)	14,923 (17.4)
Regular	8097 (47.0)	8671 (47.7)	6931 (43.1)	7031 (40.5)	6829 (40.7)	37,559 (43.9)
Smoking status, *n* (%)						
Never smoker	8027 (46.6)	10,065 (55.4)	10,334 (64.2)	12,026 (69.3)	12,186 (72.5)	52,638 (61.5)
Former smoker	4715 (27.4)	4625 (25.4)	3419 (21.3)	3159 (18.2)	2655 (15.8)	18,573 (21.7)
Current smoker	4487 (26.0)	3487 (19.2)	2334 (14.5)	2178 (12.5)	1958 (11.7)	14,444 (16.9)
Exercise habits, *n* (%)						
Almost none	7272 (42.2)	6699 (36.9)	5740 (35.7)	6517 (37.5)	6834 (40.7)	33,062 (38.6)
1–2 times a week	6627 (38.5)	7002 (38.5)	6113 (38.0)	6326 (36.4)	5867 (34.9)	31,935 (37.3)
3–5 times a week	2037 (11.8)	2757 (15.2)	2624 (16.3)	2838 (16.4)	2562 (15.3)	12,818 (15.0)
Almost everyday	1293 (7.5)	1719 (9.5)	1610 (10.0)	1682 (9.7)	1536 (9.1)	7840 (9.2)
Body mass index category, *n* (%)						
Underweight	259 (1.5)	1032 (5.7)	1614 (10.0)	2387 (13.8)	3108 (18.5)	8400 (9.8)
Normal weight	9065 (52.6)	12,991 (71.5)	12,346 (76.8)	13,590 (78.3)	12,940 (77.0)	60,932 (71.1)
Obesity/overweight	7904 (45.9)	4154 (22.9)	2127 (13.2)	1386 (8.0)	750 (4.5)	16,321 (19.1)
Laboratory measures						
Total bilirubin, mg/dl, mean (SD)	0.9 (0.3)	0.8 (0.3)	0.8 (0.3)	0.8 (0.3)	0.8 (0.3)	0.8 (0.3)
AST, IU/L, mean (SD)	26.8 (12.2)	21.5 (6.9)	20.5 (9.1)	19.5 (5.5)	19.9 (12.8)	21.7 (10.0)
ALT, IU/L, mean (SD)	42.8 (24.6)	23.4 (7.9)	18.5 (8.3)	15.1 (4.4)	12.2 (6.2)	22.5 (16.7)
γ‐GTP, IU/L, mean (SD)	61.4 (59.5)	38.0 (37.7)	28.6 (32.5)	22.3 (25.2)	19.2 (31.7)	34.0 (42.1)
ALP, IU/L, mean (SD)	206.8 (60.1)	191.7 (59.6)	183.7 (54.4)	175.7 (52.5)	168.3 (51.2)	185.4 (57.3)
AST/ALT ratio	0.7 (0.1)	0.9 (0.1)	1.1 (0.0)	1.3 (0.1)	1.7 (0.3)	1.1 (0.4)
Fatty liver, *n* (%)	8872 (51.5)	4136 (22.8)	1779 (11.1)	870 (5.0)	405 (2.4)	16,062 (18.8)
Hypertension, *n* (%)	1582 (9.2)	1600 (8.8)	1108 (6.9)	959 (5.5)	697 (4.2)	5946 (6.9)
Diabetes, *n* (%)	478 (2.8)	430 (2.4)	279 (1.7)	202 (1.2)	118 (0.7)	1507 (1.8)
Family history of cancer, *n* (%)	6309 (36.6)	7236 (39.8)	6560 (40.8)	7144 (41.1)	6834 (40.7)	34,083 (39.8)
Viral hepatitis						
Positive for HCV antibody	744 (4.4)	822 (4.6)	661 (4.2)	702 (4.1)	735 (4.5)	3664 (4.4)
Positive for HBs antigen	146 (0.9)	165 (0.9)	123 (0.8)	114 (0.7)	76 (0.5)	624 (0.8)
Positive for HBs antibody	1805 (10.7)	2285 (12.9)	2062 (13.1)	2343 (13.8)	2337 (14.3)	10,832 (13.0)

Abbreviations: ALP, alkaline phosphatase; ALT, alanine aminotransferase; AST, aspartate aminotransferase; HBs, hepatitis B surface; HCV, hepatitis C virus; γ‐GTP, γ‐glutamyl transpeptidase.

**FIGURE 1:**
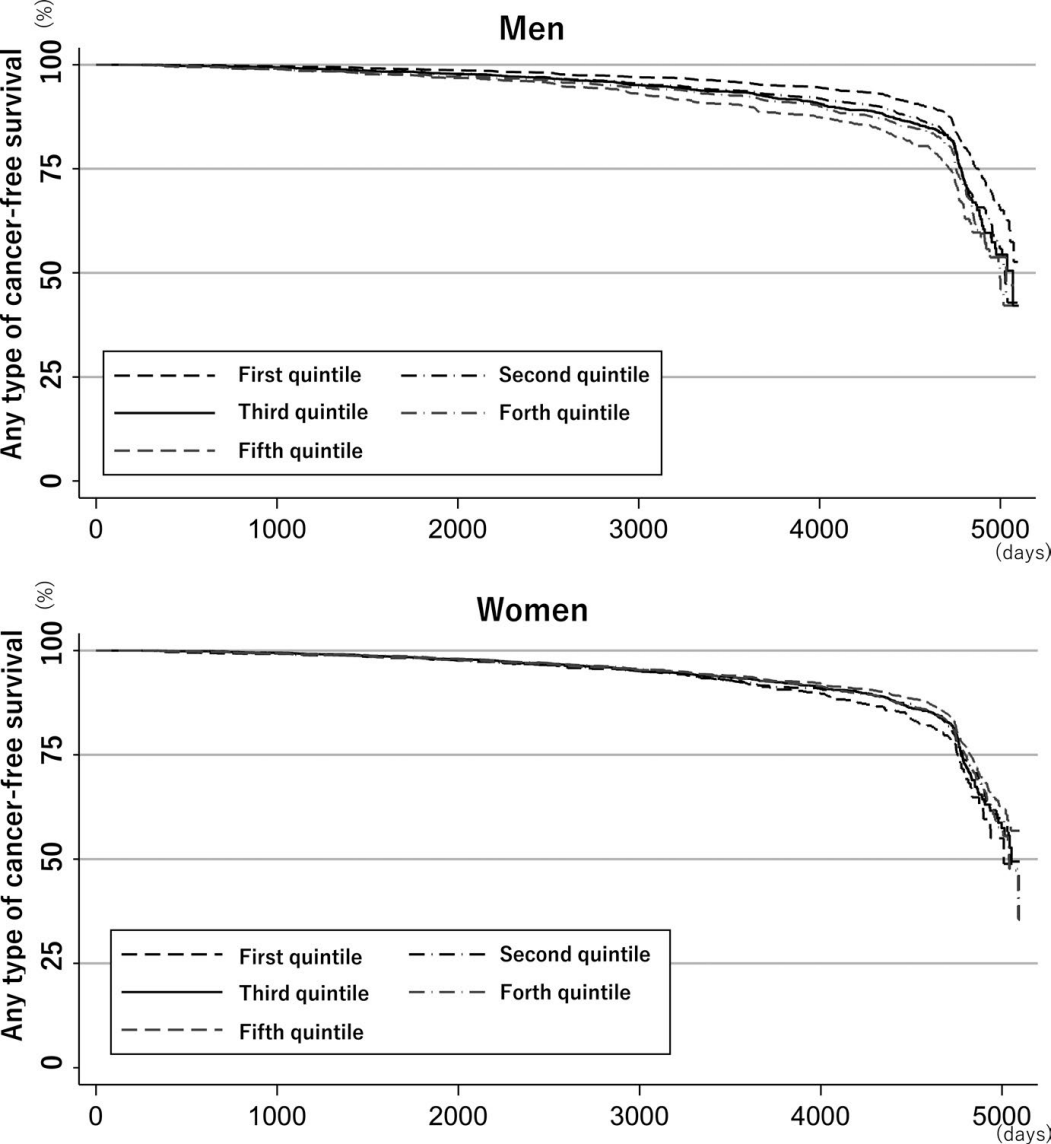
Any type of cancer‐free survival by aspartate aminotransaminase (AST)/alanine aminotransaminase (ALT) ratio category, stratified by gender

**FIGURE 2:**
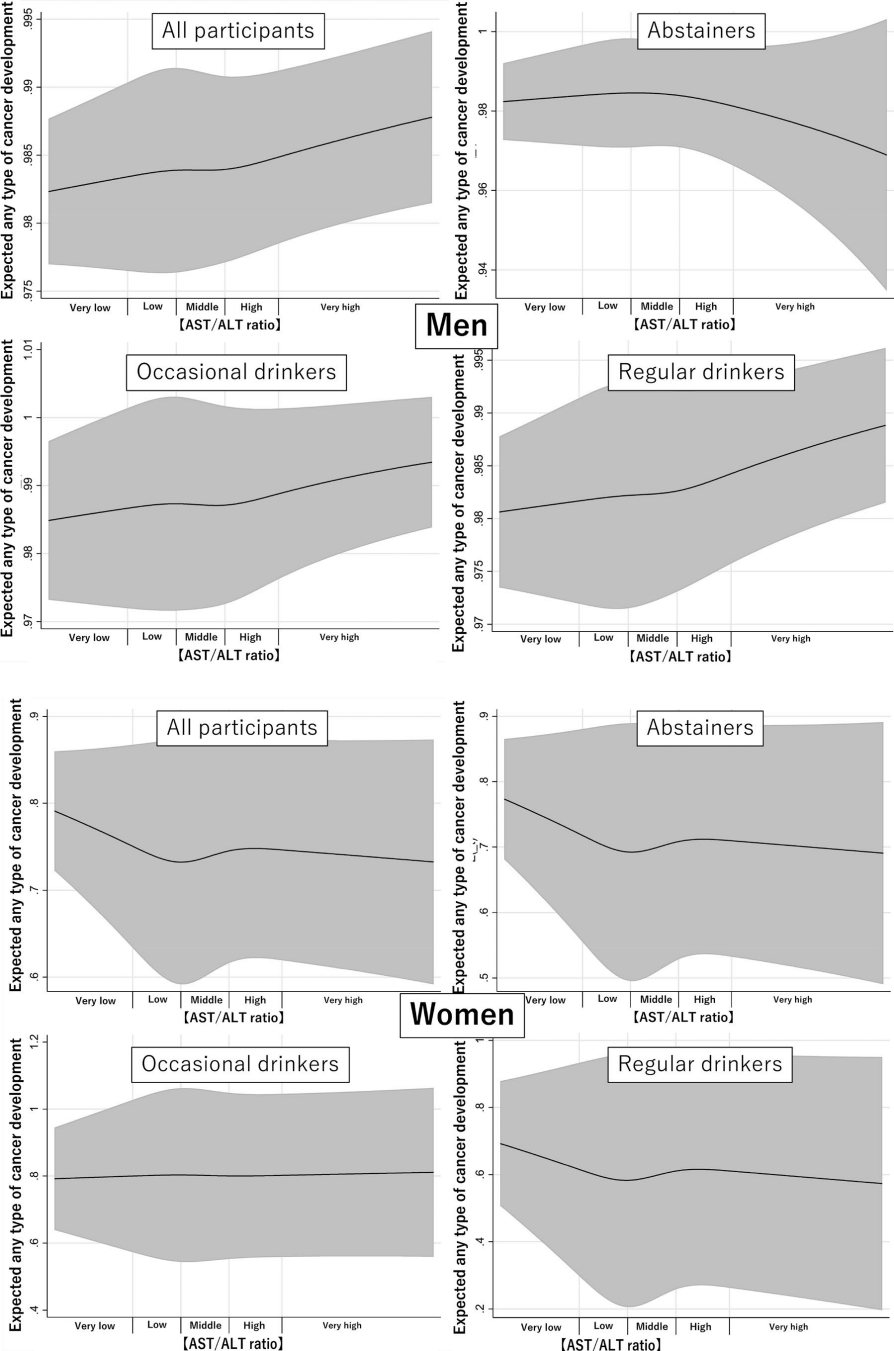
Estimated restricted cubic splines of the associations between the aspartate aminotransaminase (AST)/alanine aminotransaminase (ALT) ratios and any type of cancer, stratified sex, and alcohol consumption

During the median follow‐up of 61.6 months (interquartile range 25.4–117.7), 4701 (5.5%) patients developed some type of cancer. Effect modifications of sex (*p* < 0.01) and alcohol consumption status (*p* = 0.02) on the association between cancer development and AST/ALT ratio were significant. Tests of proportional‐hazards assumption using Schoenfeld residual among men (*p* = 0.08) and women (*p* = 0.09) did not give significant results, suggesting that the proportional‐hazard assumption was maintained. Figure 1 shows any type of cancer‐free survival by AST/ALT ratio category stratified by sex. Table 2 shows the adjusted hazard ratios (HRs) for the development of any type of cancer by AST/ALT ratio category by sex. The development of any type of cancer was not similar across all the AST/ALT ratio groups in both men and women. Among men who were abstainers, the very high AST/ALT ratio group had an increased risk for the development of any type of cancer as compared to the middle AST/ALT ratio group. Similarly, among men who were occasional drinkers, the very low AST/ALT ratio group had a lower incidence of any type of cancer development. In contrast, among men who were regular drinkers, the very high and high AST/ALT ratio groups had higher incidences of any type of cancer development. Among women, regardless of alcohol consumption, the adjusted HR for the development of any type of cancer was similar across all the AST/ALT ratio groups. Figure 2 shows the estimated restricted cubic splines of the associations between AST/ALT ratio and any type of cancer. Among men, the associations in all participants, occasional drinkers, and regular drinkers show that the risk of developing any type of cancer increased as the AST/ALT ratio increased, whereas that in abstainers shows that the risk of developing any type of cancer decreased as the AST/ALT ratio increased. In contrast, among women, the risk of developing any type of cancer decreased as the AST/ALT ratio increased, regardless of alcohol consumption. All estimated restricted cubic splines had very wide 95% confidence intervals.

**TABLE 2:** Adjusted hazard ratios for the development of any type of cancer by AST/ALT ratio category by gender

AST/ALT ratio	Number of any type of cancer development Adjusted hazard ratio (95% confidence interval)				
	Very low First quintile	Low Second quintile	Middle Third quintile	High Fourth quintile	Very high Fifth quintile
Men					
All participants	536	637	409	357	277
	0.96 (0.84–1.11)	1.03 (0.91–1.17)	Reference	1.07 (0.93–1.23)	1.17 (0.99–1.36)
Only abstainers	159	153	92	64	32
	0.94 (0.72–1.22)	0.94 (0.72–1.22)	Reference	0.83 (0.60–1.15)	**0.64 (0.42–0.97)**
Only occasional drinkers	85	81	61	45	30
	**0.69 (0.48–0.98)**	0.72 (0.52–1.01)	Reference	0.72 (0.48–1.06)	1.06 (0.68–1.66)
Only regular drinkers	292	403	256	248	215
	1.04 (0.87–1.25)	1.14 (0.97–1.33)	Reference	**1.26 (1.05–1.50)**	**1.36 (1.13–1.63)**
Women					
All participants	172	412	497	691	713
	1.16 (0.96–1.39)	1.00 (0.88–1.15)	Reference	1.03 (0.91–1.15)	1.02 (0.91–1.14)
Only abstainers	105	230	259	356	314
	1.17 (0.91–1.49)	0.97 (0.88–1.21)	Reference	1.03 (0.88–1.21)	0.98 (0.83–1.16)
Only occasional drinkers	32	86	94	120	130
	1.02 (0.67–1.56)	1.13 (0.84–1.52)	Reference	1.03 (0.79–1.36)	1.04 (0.79–1.36)
Only regular drinkers	35	96	144	215	269
	1.33 (0.90–1.95)	0.97 (0.75–1.26)	Reference	1.03 (0.83–1.28)	1.04 (0.85–1.28)

Note

Models were adjusted for age; sex; body mass index; smoking status; alcohol consumption (only for all participants); exercise habits; medical histories of hypertension, diabetes, and fatty liver; family history of any type of cancer; and the presence of hepatitis C virus (HCV) antibody, hepatitis B surface (HBs) antigen, and HBs antibody. The numbers in bold represent that the *p* value is <0.05.

Abbreviations: ALT, alanine aminotransferase; AST, aspartate aminotransferase.

Table 3 shows the adjusted HRs for the development of each type of cancer by AST/ALT ratio category among all the participants. Among men, the very high AST/ALT ratio group had higher adjusted HRs for developing esophageal cancer (adjusted HR 1.98; 95% CI 1.14–3.44), gastric cancer (adjusted HR 1.80; 95% CI 1.21–2.68), pancreatic cancer (adjusted HR 4.07; 95% CI 1.04–15.9), and cancers of the urinary tract (C64–68) as compared with the middle AST/ALT ratio group. Among men, the very low AST/ALT ratio group showed contrasting results, that is decreased esophageal cancer (adjusted HR 0.37; 95% CI 0.18–0.79) and increased urinary tract/bladder cancer (adjusted HR 2.71; 95% CI 1.02–7.18). In contrast, among women, the very high AST/ALT ratio group had neither an increased nor a decreased risk of each type of cancer. However, the very low AST/ALT ratio group in women had lower risks of cancer in the digestive organs (C15–26) (adjusted HR 0.59; 95% CI 0.38–0.94) and gastric cancer (adjusted HR 0.26; 95% CI 0.08–0.89) but had an increased risk of breast cancer (adjusted HR 1.47; 95% CI 1.11–1.95).

**TABLE 3:** Adjusted hazard ratios for the development of each type of cancer by the AST/ALT ratio category by sex

	Number of each type of cancer development Adjusted hazard ratio (95% confidence interval)									
	Men					Women				
	Very low 1st quintile	Low 2nd quintile	Middle 3rd quintile	High 4th quintile	Very high 5th quintile	Very low 1st quintile	Low 2nd quintile	Middle 3rd quintile	High 4th quintile	Very high 5th quintile
C00–C14: lips, oral cavity, and pharynx (male, 10; female, 8; mean time to development, 102.5 months)	2	4	1	1	2					
	1.86 (0.15–22.7)	2.79 (0.31–25.2)	Reference	1.29 (0.08–20.4)	3.67 (0.33–41.4)	—^a^				
C15–C26: digestive organs (male, 874; female, 453; mean time to development, 103.8 months)	226	225	151	138	134	20	80	109	122	122
	1.00 (0.80–1.25)	0.96 (0.78–1.18)	Reference	1.15 (0.91–1.44)	1.57 (1.24–1.99)	**0.59 (0.38–0.94)**	0.88 (0.65–1.18)	Reference	0.88 (0.68–1.15)	0.90 (0.70–1.17)
Esophageal cancer (male, 114; female, 31; mean time to development, 108.6 months)	11	29	24	22	28	2	4	5	9	11
	**0.37 (0.18–0.79)**	0.83 (0.48–1.43)	Reference	1.13 (0.63–2.02)	**1.98 (1.14–3.44)**	3.08 (0.59–16.2)	1.19 (0.32–4.47)	Reference	1.35 (0.45–4.05)	1.52 (0.52–4.44)
Gastric cancer (male, 306; female, 140; mean time to development, 1098.6 months)	74	77	49	54	52	3	25	32	37	43
	1.12 (0.76–1.64)	1.04 (0.72–1.49)	Reference	1.32 (0.89–1.94)	**1.80 (1.21–2.68)**	**0.26 (0.08–0.89)**	0.89 (0.52–1.52)	Reference	0.97 (0.60–1.56)	1.22 (0.76–1.94)
Duodenum cancer (male, 21; female, 12; mean time to development, 108.7 months)	6	6	3	5	1	2	3	2	2	3
	1.74 (0.41–7.39)	1.39 (0.34–5.59)	Reference	2.00 (0.48–8.40)	0.56 (0.06–5.47)	4.60 (0.59–35.8)	1.78 (0.29–10.8)	Reference	0.67 (0.09–4.81)	0.94 (0.16–5.75)
Colon cancer (male, 267; female, 166; mean time to development, 103.8 months)	84	67	50	33	33	10	32	39	45	40
	1.01 (0.69–1.47)	0.83 (0.58–1.20)	Reference	0.85 (0.54–1.32)	1.24 (0.80–1.94)	0.79 (0.38–1.65)	0.97 (0.60–1.56)	Reference	0.88 (0.57–1.36)	0.79 (0.50–1.23)
Hepatocellular carcinoma (male, 26; female, 6; mean time to development, 93.4 months)	10	8	5	2	1					
	1.58 (0.48–5.22)	1.09 (0.34–3.44)	Reference	0.51 (0.10–2.70)	0.41 (0.05–3.57)	—^a^				
Gallbladder/bile duct cancer hepatocellular carcinoma (male, 10; female, 10; mean time to development, 85.6 months)	2	3	2	1	2	0	2	4	2	2
	0.43 (0.05–3.46)	0.94 (0.15–5.78)	Reference	0.69 (0.06–7.83)	2.46 (0.31–19.3)	—^a^	0.50 (0.08–3.00)	Reference	0.44 (0.08–2.42)	0.52 (0.09–2.91)
Pancreatic cancer (male, 25; female, 15; mean time to development, 77.7 months)	5	7	3	3	7	1	1	5	4	4
	1.61 (0.36–7.24)	1.71 (0.44–6.64)	Reference	1.23 (0.25–6.13)	**4.07**	1.29 (0.15–11.2)	0.30 (0.04–2.60)	Reference	0.63 (0.17–2.37)	0.69 (0.18–2.59)
					**(1.04–15.9)**					
C30–C39: respiratory and intrathoracic organs (male, 255; female, 196; mean time to development, 109.7 months)	58	72	50	49	26	13	36	49	45	53
	0.85 (0.56–1.27)	0.95 (0.66–1.36)	Reference	**0.71 (0.51–0.98)**	0.83 (0.60–1.15)	1.20 (0.63–2.28)	0.90 (0.58–1.39)	Reference	0.68 (0.45–1.02)	0.82 (0.55–1.22)
Lung cancer (male, 217; female, 174; mean time to development, 109.6 months)	50	62	43	39	23	11	33	44	40	46
	0.87 (0.56–1.35)	0.96 (0.64–1.42)	Reference	1.12 (0.72–1.72)	0.93 (0.56–1.55)	1.12 (0.56–2.23)	0.94 (0.59–1.48)	Reference	0.67 (0.44–1.03)	0.79 (0.52–1.20)
C43–C44: melanoma and other skin (male, 14; female, 20; mean time to development, 89.3 months)	1	6	4	2	1	0	7	2	5	6
	0.91 (0.05–18.0)	0.65 (0.04–11.2)	Reference	—^a^	—^a^	—^a^	—^a^	Reference	1.33 (0.12–14.8)	1.08 (0.10–12.1)
C50–C50: breast tissue (male, 2; female, 993; mean time to development, 110.6 months)						78	162	180	287	286
	—^a^					**1.47 (1.11–1.95)**	1.08 (0.87–1.35)	Reference	1.14 (0.95–1.38)	1.03 (0.86–1.25)
C51–C58: female genital organs (female, 459; mean time to development, 99.4 months)						32	71	76	136	144
	—^a^					1.34 (0.86–2.08)	1.17 (0.84–1.62)	Reference	1.27 (0.96–1.69)	1.24 (0.94–1.65)
Cervical cancer (female, 166; mean time to development, 92.7 months)						6	22	28	47	63
	—^a^					0.75 (0.30–1.87)	1.02 (0.58–1.78)	Reference	1.14 (0.71–1.82)	1.27 (0.81–1.99)
Endometrial cancer (female, 96; mean time to development, 109.4 months)						6	18	13	32	27
	—^a^					1.53 (0.55–4.23)	1.70 (0.83–3.48)	Reference	1.83 (0.96–3.49)	1.50 (0.77–2.92)
Ovarian cancer (female, 63; mean time to development, 92.5 months)						10	8	15	14	16
	—^a^					2.26 (0.94–5.41)	0.68 (0.28–1.60)	Reference	0.68 (0.33–1.41)	0.73 (0.36–1.48)
C60–C63: male genital organs (male, 700; mean time to development, 113.9 months)	157	221	138	107	77					
	1.07 (0.84–1.38)	1.13 (0.91–1.40)	Reference	0.88 (0.68–1.13)	0.86 (0.65–1.14)	—^a^				
Prostate cancer (male, 624; mean time to development, 112.0 months)	133	196	126	99	70					
	1.05 (0.81–1.37)	1.11 (0.88–1.39)	Reference	0.88 (0.67–1.15)	0.84 (0.62–1.13)	—^a^				
C64–C68: urinary tract (male, 107; female, 59; mean time to development, 107.3 months)	29	29	10	26	13	3	13	16	12	15
	1.86 (0.87–3.97)	1.83 (0.89–3.78)	Reference	**3.19 (1.54–6.64)**	**2.47 (1.07–5.67)**	0.56 (0.15–2.04)	0.90 (0.42–1.89)	Reference	0.59 (0.28–1.26)	0.77 (0.38–1.74)
Renal cancer (male, 42; female, 29; mean time to development, 112.4 months)	12	11	4	9	6	1	8	5	7	8
	1.07 (0.32–3.54)	1.44 (0.45–4.55)	Reference	3.01 (0.92–9.82)	3.40 (0.95–12.2)	0.62 (0.07–5.74)	1.77 (0.57–5.48)	Reference	1.06 (0.34–3.37)	1.22 (0.39–3.76)
Urinary tract/bladder cancer (male, 65; female, 30; mean time to development, 103.6 months)	17	18	6	17	7	2	5	11	5	7
	**2.71 (1.02–7.18)**	2.16 (0.85–5.46)	Reference	**3.32 (1.30–8.44)**	1.94 (0.65–5.84)	0.53 (0.11–2.65)	0.48 (0.16–1.42)	Reference	0.35 (0.12–1.03)	0.53 (0.20–1.40)
C73–C75: thyroid and other endocrine glands (male, 9; female, 31; mean time to development, 97.3 months)						4	4	6	10	7
	—^a^					2.20 (0.57–8.54)	0.80 (0.22–2.85)	Reference	1.27 (0.46–3.51)	0.90 (0.30–2.68)
C76–C80: ill‐defined, other secondary and unspecified sites (male, 18; female, 20; mean time to development, 96.9 months)	3	5	4	4	2	1	4	6	3	6
	0.39 (0.08–1.93)	0.78 (0.21–2.93)	Reference	1.30 (0.32–5.21)	1.04 (0.19–5.74)	0.84 (0.09–7.61)	0.81 (0.22–2.91)	Reference	0.38 (0.09–1.53)	0.66 (0.21–2.07)
C81–C96: lymphoid, hematopoietic, and related tissues (male, 58; female, 52; mean time to development, 99.1 months)	13	23	14	4	4	2	5	16	18	11
	0.52 (0.23–1.18)	1.02 (0.52–2.00)	Reference	0.37 (0.12–1.14)	0.53 (0.17–1.63)	0.47 (0.10–2.17)	0.38 (0.14–1.06)	Reference	0.84 (0.43–1.65)	0.52 (0.24–1.14)
Lymphoma (male, 35; female, 42; mean time to development, 97.0 months)	7	15	7	3	3	1	3	13	17	8
	0.71 (0.23–2.18)	1.46 (0.59–3.60)	Reference	0.54 (0.14–2.11)	0.70 (0.18–2.75)	0.28 (0.04–2.28)	**0.27 (0.08–0.96)**	Reference	0.99 (0.48–2.05)	0.49 (0.20–1.19)
Leukemia (male, 14; female, 8; mean time to development, 105.0 months)	4	4	5	1	0					
	0.33 (0.08–1.41)	0.43 (0.11–1.62)	Reference	0.26 (0.03–2.27)	—^a^	—^a^				
D00–D09: in situ neoplasms (male, 8; female, 74; mean time to development, 90.7 months)						5	12	13	22	22
	—^a^					1.31 (0.44–3.88)	1.19 (0.54–2.63)	Reference	1.13 (0.57–2.24)	0.93 (0.47–1.86)
Unknown (male, 182; female, 125; mean time to development, 97.3 months)	49	55	34	27	17	14	22	26	27	36
	0.97 (0.60–1.57)	1.05 (0.68–1.64)	Reference	1.03 (0.61–1.74)	0.97 (0.53–1.75)	**2.11 (1.05–4.21)**	1.05 (0.59–1.87)	Reference	0.75 (0.44–1.29)	1.02 (0.62–1.71)

Note

Models were adjusted for age; sex; body mass index; smoking status; alcohol consumption; exercise habits; medical histories of hypertension, diabetes, fatty liver; family history of any type of cancer; and, the presence of hepatitis C virus (HCV) antibody, hepatitis B surface (HBs) antigen, and HBs antibody. The following cancers which had less than 10 cases in each sex were omitted from the analyses due to the lack of statistical power. The numbers in bold represent that the *p* value is <0.05.

Abbreviations: ALT, alanine aminotransferase; AST, aspartate aminotransferase; C40–C41, bone and articular cartilage (male, 0; C45–C49, mesothelial and soft tissue [male, 3; C69–C72, eye, brain, and other parts of the central nervous system {male, 1; female, 1}; female, 1]; female, 5).

^a^ Hazard ratios could not be calculated due to an insufficient number of events.

Table 4 shows the adjusted HRs for the development of each type of cancer by AST/ALT ratio category stratified by alcohol consumption status and sex. The findings were similar to those in Table 3, but they were remarkable in regular drinkers.

**TABLE 4:** Adjusted hazard ratios for the development of each type of cancer by the AST/ALT ratio category

	Number of each type of cancer development Adjusted hazard ratios (95% confidence interval)														
	Abstainers					Occasional drinkers					Regular drinkers				
	First	Second	Third	Fourth	Fifth	First	Second	Third	Fourth	Fifth	First	Second	Third	Fourth	Fifth
Men															
C15–C26	63	43	28	19	12	30	25	20	10	15	133	157	103	109	107
	0.96 (0.59–1.56)	0.79 (0.49–1.28)	Reference	0.77 (0.43–1.39)	0.75 (0.37–1.54)	0.70 (0.38–1.30)	0.69 (0.38–1.25)	Reference	0.51 (0.24–1.11)	1.68 (0.84–3.35)	1.05 (0.80–1.38)	1.06 (0.83–1.37)	Reference	**1.38** (**1.06–1.81**)	**1.72** (**1.31–2.27)**
Esophageal cancer	2	2	3	2	2						9	25	18	19	26
	0.56 (0.08–3.75)	0.31 (0.05–1.98)	Reference	0.46 (0.07–3.10)	0.97 (0.13–7.10)	—^a^					**0.41 (0.18–0.94)**	0.96 (0.52–1.76)	Reference	1.35 (0.70–2.57)	**2.46 (1.33–4.54)**
Gastric cancer	23	15	12	8	5	14	8	8	5	6	37	54	29	41	41
	1.07 (0.49–2.30)	0.62 (0.28–1.35)	Reference	0.56 (0.22–1.41)	0.55 (0.18–1.68)	0.59 (0.23–1.50)	0.49 (0.18–1.33)	Reference	0.57 (0.18–1.79)	1.91 (0.63–5.75)	1.20 (0.72–2.00)	1.37 (0.87–2.16)	Reference	**1.81 (1.12–2.91)**	**2.20 (1.36–3.57)**
Colon cancer	26	15	7	6	3	9	6	6	4	8	49	46	37	23	22
	1.28 (0.52–3.17)	1.11 (0.45–2.75)	Reference	1.18 (0.39–3.51)	1.00 (0.26–3.92)	0.93 (0.31–2.80)	0.59 (0.19–1.85)	Reference	0.64 (0.18–2.32)	2.44 (0.82–7.23)	0.99 (0.62–1.56)	0.83 (0.54–1.29)	Reference	0.82 (0.49–1.39)	1.04 (0.61–1.77)
C30–C39	21	19	9	8	2	6	11	7	7	4	31	42	34	34	20
	1.01 (0.43–2.35)	1.10 (0.49–2.46)	Reference	1.02 (0.39–2.69)	0.48 (0.10–2.31)	0.36 (0.11–1.16)	0.81 (0.31–2.11)	Reference	1.02 (0.35–2.96)	1.06 (0.30–3.78)	0.92 (0.55–1.55)	0.93 (0.59–1.47)	Reference	1.31 (0.81–2.12)	0.90 (0.51–1.59)
Lung cancer	18	15	8	5	2	6	9	5	6	4	26	38	30	28	17
	1.04 (0.42–2.56)	0.97 (0.40–2.31)	Reference	0.71 (0.23–2.20)	0.57 (0.12–2.74)	0.52 (0.15–1.84)	0.91 (0.30–2.74)	Reference	1.16 (0.34–3.88)	1.44 (0.37–5.68)	0.86 (0.49–1.51)	0.95 (0.59–1.55)	Reference	1.23 (0.73–2.06)	0.90 (0.49–1.65)
C60–C63	46	59	38	23	12	30	29	23	17	8	81	133	77	67	57
	0.82 (0.51–1.33)	0.91 (0.60–1.39)	Reference	0.65 (0.38–1.10)	**0.46 (0.23–0.90)**	0.77 (0.20–3.03)	0.58 (0.14–2.34)	Reference	1.23 (0.32–4.72)	0.50 (0.05–4.78)	1.28 (0.91–1.78)	**1.39 (1.04–1.84)**	Reference	1.11 (0.80–1.55)	1.12 (0.79–1.59)
Prostate cancer	41	53	35	20	12	27	20	21	16	8	65	**123**	70	63	50
	0.79 (0.48–1.30)	0.87 (0.56–1.34)	Reference	0.59 (0.34–1.04)	**0.50 (0.25–0.99)**	0.82 (0.44–1.52)	**0.52 (0.28–0.98)**	Reference	0.65 (0.33–1.27)	0.73 (0.31–1.70)	1.22 (0.85–1.75)	**1.45 (1.08–1.95)**	Reference	1.15 (0.82–1.62)	1.08 (0.75–1.56)
C64–C68	9	7	1	6	2	6	4	4	5	1	14	18	5	15	10
	3.89 (0.46–32.9)	3.86 (0.47–31.6)	Reference	7.09 (0.84–59.8)	5.26 (0.47–58.6)	1.21 (0.31–4.64)	1.14 (0.30–4.36)	Reference	3.06 (0.89–10.5)	0.32 (0.03–3.00)	2.21 (0.77–6.39)	2.42 (0.89–6.55)	Reference	**3.95** (**1.43–10.9**)	**3.35** (**1.13–9.92**)
Kidney cancer	3	5	1	2	1	2	2	1	4	0	7	4	2	3	5
	0.88 (0.08–9.86)	2.60 (0.30–22.7)	Reference	2.54 (0.23–28.3)	2.34 (0.14–38.3)	0.54 (0.04–7.02)	1.05 (0.09–12.0)	Reference	5.46 (0.55–54.1)	—^a^	1.48 (0.29–7.63)	1.03 (0.19–5.66)	Reference	2.09 (0.35–12.6)	4.86 (0.92–25.8)
Urinary tract/bladder cancer						4	2	3	1	1	7	14	3	12	5
	—^a^					1.16 (0.23–5.91)	0.50 (0.08–3.06)	Reference	0.30 (0.03–2.97)	0.71 (0.07–7.63)	2.61 (0.65–10.6)	**3.57 (1.02–12.5)**	Reference	**5.20** (**1.46–18.5**)	2.64 (0.62–11.2)
C81–C96	3	9	3	0	2	3	2	2	3	0	7	12	9	1	2
	0.43 (0.08–2.46)	1.66 (0.43–6.40)	Reference	—^a^	0.96 (0.14–6.61)	1.02 (0.15–6.87)	0.60 (0.08–4.28)	Reference	1.56 (0.25–9.58)	—^a^	0.44 (0.15–1.28)	0.83 (0.34–1.99)	Reference	0.16 (0.02–1.29)	0.42 (0.09–1.96)
Unknown	16	10	10	6	2	7	11	2	2	1	26	34	22	19	14
	0.91 (0.36–2.32)	0.65 (0.25–1.70)	Reference	1.01 (0.35–2.92)	0.55 (0.11–2.60)	1.07 (0.20–5.62)	2.47 (0.54–11.4)	Reference	1.19 (0.16–8.57)	1.55 (0.14–17.3)	1.01 (0.55–1.84)	1.08 (0.63–1.85)	Reference	1.03 (0.55–1.92)	1.06 (0.53–2.09)
Women															
C15–C26	11	44	60	69	61	3	20	19	20	15	6	16	30	33	46
	**0.48 (0.24–0.95)**	0.79 (0.53–1.18)	Reference	0.92 (0.65–1.31)	0.91 (0.63–1.31)	0.61 (0.17–2.15)	1.37 (0.71–2.64)	Reference	0.92 (0.49–1.76)	0.70 (0.35–1.41)	1.00 (0.40–2.51)	0.76 (0.41–1.40)	Reference	0.78 (0.47–1.28)	0.92 (0.58–1.47)
Esophagus cancer	0	2	2	5	4						2	0	1	2	6
	—^a^	1.39 (0.19–10.1)	Reference	1.96 (0.37–10.4)	1.31 (0.22–7.75)	—^a^					**18.1 (1.51–217)**	—^a^	Reference	1.34 (0.12–15.0)	2.97 (0.35–25.1)
Gastric cancer	**1**	15	23	21	28	1	7	3	5	6	1	3	6	11	9
	**0.10 (0.01–0.76)**	0.66 (0.34–1.30)	Reference	0.76 (0.42–1.38)	1.18 (0.67–2.07)	1.69 (0.15–18.8)	4.26 (0.91–20.0)	Reference	2.27 (0.46–11.2)	2.95 (0.62–14.1)	1.03 (0.11–9.27)	0.69 (0.17–2.84)	Reference	1.36 (0.50–3.71)	0.96 (0.34–2.71)
Colon cancer	6	17	15	25	17	2	6	11	8	4	2	9	13	12	19
	1.10 (0.40–2.99)	1.24 (0.61–2.51)	Reference	1.30 (0.68–2.47)	0.98 (0.48–1.97)	0.54 (0.11–2.71)	0.64 (0.23–1.80)	Reference	0.60 (0.24–1.50)	**0.30 (0.09–0.95)**	0.54 (0.11–2.58)	0.94 (0.39–2.22)	Reference	0.63 (0.29–1.40)	0.88 (0.43–1.80)
C30–C39	9	22	30	28	32	1	7	6	5	8	3	7	13	12	13
	1.29 (0.59–2.81)	0.87 (0.50–1.53)	Reference	0.71 (0.42–1.19)	0.88 (0.53–1.46)	0.81 (0.09–6.95)	1.47 (0.48–4.50)	Reference	0.68 (0.20–2.28)	1.26 (0.43–3.71)	1.34 (0.36–5.02)	0.68 (0.26–1.83)	Reference	0.69 (0.31–1.53)	0.67 (0.31–1.47)
Lung cancer	7	21	26	25	30	1	7	6	5	5	3	5	12	10	11
	1.17 (0.49–2.79)	0.96 (0.54–1.72)	Reference	0.73 (0.42–1.26)	0.94 (0.55–1.60)	0.83 (0.10–7.17)	1.45 (0.47–4.48)	Reference	0.67 (0.20–2.24)	0.79 (0.24–2.64)	1.40 (0.37–5.35)	0.61 (0.21–1.77)	Reference	0.63 (0.27–1.48)	0.62 (0.27–1.44)
C50–C50	46	85	87	136	108	14	32	35	56	53	18	45	58	95	125
	**1.52 (1.03–2.22)**	1.03 (0.76–1.40)	Reference	1.12 (0.86–1.47)	0.93 (0.70–1.24)	1.13 (0.58–2.17)	1.14 (0.70–1.86)	Reference	1.30 (0.85–1.99)	1.08 (0.70–1.67)	**1.79 (1.02–3.14)**	1.11 (0.74–1.65)	Reference	1.12 (0.81–1.56)	1.15 (0.83–1.57)
C51–C58	19	39	35	73	60	8	12	19	18	31	5	20	22	45	53
	1.43 (0.78–2.60)	1.24 (0.78–1.97)	Reference	**1.53 (1.02–2.30)**	1.32 (0.87–2.02)	1.25 (0.52–2.99)	0.80 (0.39–1.67)	Reference	0.74 (0.39–1.42)	1.08 (0.61–1.93)	1.27 (0.46–3.50)	1.40 (0.76–2.60)	Reference	1.34 (0.79–2.27)	1.22 (0.73–2.03)
Cervical cancer	4	10	10	21	26	2	2	10	7	15	0	10	8	19	22
	1.16 (0.34–3.92)	1.16 (0.48–2.81)	Reference	1.50 (0.71–3.19)	1.81 (0.87–3.78)	0.59 (0.12–2.99)	0.25 (0.05–1.17)	Reference	0.53 (0.20–1.41)	0.88 (0.39–1.98)	—^a^	1.89 (0.74–4.82)	Reference	1.48 (0.65–3.39)	1.17 (0.52–2.65)
Endometrial cancer	5	7	8	21	12	0	5	2	4	8	1	6	3	7	7
	1.50 (0.44–5.09)	0.97 (0.35–2.70)	Reference	2.06 (0.91–4.66)	1.30 (0.53–3.20)	—^a^	2.93 (0.56–15.4)	Reference	1.34 (0.24–7.41)	2.95 (0.62–14.1)	2.32 (0.23–23.8)	2.93 (0.73–11.8)	Reference	1.48 (0.38–5.77)	1.13 (0.29–4.39)
Ovarian cancer	5	5	9	8	9	3	1	1	3	1	2	2	5	3	6
	1.92 (0.59–6.22)	0.68 (0.23–2.06)	Reference	0.64 (0.25–1.66)	0.74 (0.29–1.87)	8.10 (0.76–86.2)	1.36 (0.08–22.1)	Reference	2.44 (0.25–23.7)	0.78 (0.05–12.7)	1.36 (0.22–8.45)	0.55 (0.10–2.91)	Reference	0.45 (0.11–1.91)	0.77 (0.23–2.59)
C64–C68	1	9	11	4	8	1	2	3	4	3	1	2	2	4	4
	0.26 (0.03–2.17)	0.90 (0.36–2.21)	Reference	**0.29** (**0.09–0.90**)	0.60 (0.24–1.52)	0.73 (0.06–8.71)	0.46 (0.07–3.15)	Reference	1.30 (0.27–6.20)	0.99 (0.19–5.17)	3.12 (0.24–41.0)	1.30 (0.18–9.49)	Reference	1.56 (0.28–8.71)	1.36 (0.24–7.62)
Kidney cancer	1	6	4	3	5										
	0.98 (0.10–9.28)	1.73 (0.48–6.21)	Reference	0.54 (0.12–2.45)	0.93 (0.25–3.51)	—^a^					—^a^				
Urinary tract/bladder cancer	0	3	7	1	3	1	2	2	2	1					
	—^a^	0.38 (0.09–1.59)	Reference	**0.10** (**0.01–0.87**)	0.34 (0.08–1.46)	1.79 (0.12–26.9)	0.82 (0.10–7.04)	Reference	0.85 (0.11–6.84)	0.40 (0.03–5.10)	—^a^				
C81–C96	2	5	8	11	6						0	0	4	4	3
	1.06 (0.22–5.24)	0.71 (0.23–2.19)	Reference	0.98 (0.39–2.45)	0.59 (0.20–1.73)	—^a^					—^a^	—^a^	Reference	0.70 (0.17–2.89)	0.49 (0.10–2.28)
Lymphoma	1	3	7	10	4						0	0	3	4	2
	0.65 (0.08–5.44)	0.47 (0.12–1.85)	Reference	1.00 (0.38–2.65)	0.47 (0.13–1.63)	—^a^					—^a^	—^a^	Reference	0.70 (0.17–2.89)	0.49 (0.10–2.28)
D00–D09	3	6	8	13	8	1	2	2	2	8	1	4	3	7	6
	1.02 (0.25–4.19)	0.86 (0.30–2.49)	Reference	1.11 (0.46–2.69)	0.62 (0.23–1.68)	1.59 (0.13–19.7)	1.29 (0.17–9.56)	Reference	0.70 (0.10–5.00)	2.48 (0.50–11.4)	2.60 (0.27–25.5)	2.11 (0.47–9.54)	Reference	1.58 (0.40–6.19)	0.97 (0.24–3.97)
Unknown	9	15	16	13	16	4	6	5	6	6	1	1	5	8	14
	1.84 (0.76–4.42)	1.06 (0.52–2.16)	Reference	0.56 (0.26–1.18)	0.82 (0.41–1.65)	3.06 (0.75–12.4)	1.35 (0.40–4.50)	Reference	0.97 (0.29–3.21)	0.92 (0.28–3.04)	1.48 (0.16–13.4)	0.33 (0.04–2.83)	Reference	1.11 (0.36–3.41)	1.53 (0.55–4.29)

Note

Models were adjusted for age; sex; body mass index; smoking status; alcohol consumption; exercise habits; medical histories of hypertension, diabetes, and fatty liver; family history of any type of cancer; and, the presence of hepatitis C virus (HCV) antibody, hepatitis B surface (HBs) antigen, and HBs antibody. The following cancers which had less than 10 cases in each sex/alcohol consumption status were omitted from the analyses due to the lack of statistical power. The numbers in bold represent that the *p* value is <0.05.

Abbreviations: ALT, alanine aminotransferase; AST, aspartate aminotransferase; C00–C14, lips, oral cavity, and pharynx; C40–C41, bone and articular cartilage; C43–C44, melanoma and other skin cancers; C45–C49, mesothelial and soft tissue; C69–C72, eye, brain, and other parts of the central nervous system; C73–C75, thyroid and other endocrine glands; C76–C80, ill‐defined, other secondary, and unspecified sites; duodenal cancer; gallbladder/bile duct cancer hepatocellular carcinoma; hepatocellular carcinoma; leukemiapancreatic cancer.

^a^ Hazard ratios could not be calculated due to an insufficient number of events.

Supporting Information 1 shows the results of the sensitivity analyses excluding participants with fatty liver, and Supporting Information 2 shows those excluding high AST and ALT levels. All the adjusted HRs from the sensitivity analyses were fairly similar to those in the main results.

## 4 DISCUSSION

Men with higher AST/ALT ratios tended to have higher risks of developing any type of cancer in the future as compared to those with middle AST/ALT ratios, especially among regular drinkers. In contrast, women did not show any association between the development of any type of cancer and the AST/ALT ratio, regardless of alcohol consumption. Among men, the participants with higher AST/ALT ratios had higher risks of esophageal, gastric and pancreatic and urinary tract cancers, but lower risks of cancers in the respiratory and intrathoracic organs as compared with those with middle AST/ALT ratios. Among women, the participants with very low AST/ALT ratios had higher risks of breast cancer as compared to those with middle AST/ALT ratios.

We hypothesized that the following two underlying mechanisms influenced the association between the AST/ALT ratio and cancer development: the lifestyle‐related mechanism and the pathophysiological mechanism. People who consumed alcohol regularly were known to have higher AST/ALT ratios.^14^ Although we have adjusted for or stratified by alcohol consumption status for the analysis, we might not be able to consider the exact or time‐varying amount of alcohol consumption in the analysis. On the contrary, the AST/ALT ratio increases after exercise.^15^, ^16^ Similarly, eating quickly may be associated with a lower AST/ALT ratio.^17^ In fact, a previous study reported that a high ALT/AST ratio may be a good surrogate marker for insulin resistance,^18^ suggesting the influence of lifestyle. Such unmeasured lifestyles may be associated with the AST/ALT ratio, resulting in differences in the future risk of cancer development in our study.

Another hypothetical mechanism for the association is the pathophysiological mechanism. ALT is a more liver‐specific measurement than AST.^19^ People who experience hepatic aging, indicated by low ALT levels, may produce more free radicals and oxidative stress,^12^ causing cancer. In addition, a previous study reported lower ALT levels in the sera of patients with invasive cancer cells.^20^ People who have occult cancer at baseline, causing low ALT levels, may be diagnosed with cancer during the follow‐up. By contrast, AST is known to be an enzyme released from different tissues, reflecting anaerobic glycolysis.^21^ People with increased AST levels may have higher cancer proliferation rates and more severe tissue damage.^8^ As a result, decreased ALT and increased AST levels, causing a high AST/ALT ratio, may be related to the increased risk of cancer development through the pathophysiological mechanism. In addition, the aldehyde dehydrogenase (ALDH) 2 genotype may play a role in the pathophysiological mechanism of the association. The ALDH2 gene rs671 polymorphism was associated with the overall cancer risk in the Asian population.^22^ Another study reported that those with the ALDH2 gene rs671 polymorphism had increased AST/ALT ratios as they consumed higher amounts of alcohol.^23^ Participants with this gene have higher AST/ALT ratios than those without the gene although they consumed similar amounts of alcohol, thus increasing the risk of developing cancer due to the presence of this gene.

In terms of cancer development in regions other than the digestive organs, our study found associations between the ASL/ALT ratio and cancers in the urinary tract, respiratory organs, and the breast. Although no previous study has evaluated the association between the current AST/ALT ratio and future cancer development, some studies have evaluated the association between the AST/ALT ratio and the existence of cancer or the prognosis of patients with cancer. In fact, an elevated AST/ALT ratio was found to be associated with worsening survival in patients with upper tract urothelial cancer^24^ but not in those with renal cell carcinoma.^25^ Another study reported that an increased AST/ALT ratio may suggest the existence of prostate cancer.^10^ In terms of cancer development in the respiratory organs, to the best of our knowledge, no previous study has evaluated the association of AST/ALT ratio with the existence or the prognosis of cancer. These previous studies might support our findings; that is, there is a possible association between the AST/ALT ratio and the development of urinary tract cancers.

There are some strengths of this study. First, our study included a large population of more than 80,000 participants. This large sample size provided enough power for analysis. In addition, our study considered several factors, such as environmental risk factors and laboratory measures, which may be potential confounders. The information would be useful to minimize biases of the true association.

Our study had some limitations. First, multiple testing problems possibly occurred, especially during the sub‐analyses of each type of cancer stratified by the alcohol consumption status. Although significant differences were no longer observed in some cases after Bonferroni correction, our findings may prompt the conduct of future studies to evaluate the abovementioned association. Second, some of the participants may have had an existing occult cancer at baseline. However, based on the cumulative incidence of cancers, the number of patients diagnosed with cancer at baseline was limited. In addition, our data lacked potential important confounders, such as the use of lipid‐lowering drugs or hormone replacement therapy, which may influence the development of steatohepatitis and liver function. This unobtained data may bias the results. Moreover, our study population may have higher health awareness compared to the general population. Because the health check‐ups are completely voluntary, the participants may have had higher socioeconomic statuses. Although our study followed up participants for a median of 61.6 months, the period may have been insufficient to catch up on cancer cases.

## 5 CONCLUSION

People who had higher AST/ALT ratios tended to have higher risks of developing any type of cancer among men who were regular drinkers, but the risk was lower among men who were abstainers. Among men who were occasional drinkers, a lower AST/ALT ratio was associated with a lower risk of developing any type of cancer. Among women, regardless of alcohol consumption, there was no association between the development of any type of cancer and the AST/ALT ratio.

## CONFLICT OF INTEREST

None.

## AUTHOR CONTRIBUTIONS

Daiki Kobayashi organized and led this study, including data analysis, interpretation of result, and preparing manuscript; Kazuki Yamamoto and Takeshi Kimura contributed to manuscript preparation and data analysis; Takuro Shimbo supervised this study and contributed to manuscript preparation.

## Supporting information

Supplementary Material

Supplementary Material

## Data Availability

Due to the nature of this research, participants’ data was not shared publicly due to participants’ privacy, so supporting data is not available.
