# Supplementary material for: Aspartate aminotransferase/alanine aminotransferase ratio and subsequent cancer development
Source: Cancer Med. 2021 Dec 1;11(3):798–814. doi: 10.1002/cam4.4473 (PMC8817090; doi:10.1002/cam4.4473)
Supplement: Supplementary file 1 — Supplementary Material [file CAM4-11-798-s001.docx]

Supporting information 1. Adjusted hazard ratios for the development of any type of cancer by aspartate transaminase (AST)/alanine transaminase (ALT) ratio category by gender, excluding participants with fatty liver

|  | Number of any type of cancer development  Adjusted hazard ratios  (95% confidence interval) | | | | |
| --- | --- | --- | --- | --- | --- |
| AST/ALT ratio | Very low  First quintile | Low  Second quintile | Middle  Third quintile | High  Fourth quintile | Very high  Fifth quintile |
| Men |  |  |  |  |  |
| All participants | 219 | 436 | 311 | 309 | 248 |
|  | 1.00 | 1.15 | Reference | 1.15 | **1.22** |
|  | (0.85–1.17) | (0.98–1.34) |  | (0.96–1.36) | **(1.02–1.46)** |
| Only abstainers | 65 | 99 | 74 | 54 | 28 |
|  | 0.98 | 1.06 | Reference | 0.70 | 0.72 |
|  | (0.71–1.35) | (0.77–1.46) |  | (0.46–1.07) | (0.44–1.17) |
| Only occasional drinkers | 25 | 61 | 49 | 39 | 30 |
|  | 0.77 | 1.19 | Reference | 1.00 | 1.48 |
|  | (0.51–1.18) | (0.79–1.79) |  | (0.60–1.64) | (0.89–2.46) |
| Only regular drinkers | 129 | 276 | 188 | 216 | 190 |
|  | 1.05 | 1.17 | Reference | **1.34** | **1.35** |
|  | (0.86–1.28) | (0.96–1.42) |  | **(1.08–1.66)** | **(1.09–1.68)** |
| Women |  |  |  |  |  |
| All participants | 92 | 338 | 457 | 667 | 702 |
|  | 1.12 | 0.97 | Reference | 1.08 | 1.00 |
|  | (0.96–1.32) | (0.85–1.11) |  | (0.96–1.22) | (0.89–1.13) |
| Only abstainers | 55 | 187 | 232 | 342 | 308 |
|  | 1.19 | 0.90 | Reference | 1.04 | 0.98 |
|  | (0.96–1.47) | (0.75–1.09) |  | (0.88–1.23) | (0.82–1.16) |
| Only occasional drinkers | 17 | 66 | 90 | 115 | 128 |
|  | 1.01 | 0.91 | Reference | 1.05 | 0.90 |
|  | (0.70–1.45) | (0.67–1.24) |  | (0.79–1.40) | (0.67–1.20) |
| Only regular drinkers | 20 | 85 | 135 | 210 | 266 |
|  | 1.02 | 1.14 | Reference | 1.16 | 1.09 |
|  | (0.72–1.42) | (0.89–1.46) |  | (0.93–1.45) | (0.88–1.35) |

Models were adjusted for age; sex; body mass index; smoking status; alcohol consumption (only for all participants); exercise habits; medical histories of hypertension, and diabetes; family history of any type of cancer; and, the presence of hepatitis C virus (HCV) antibody, hepatitis B surface (HBs) antigen, and HBs antibody.

The numbers in bold represent that the p value is <0.05.
