# Supplementary material for: Aspartate aminotransferase/alanine aminotransferase ratio and subsequent cancer development
Source: Cancer Med. 2021 Dec 1;11(3):798–814. doi: 10.1002/cam4.4473 (PMC8817090; doi:10.1002/cam4.4473)
Supplement: Supplementary file 2 — Supplementary Material [file CAM4-11-798-s002.docx]

Supporting information 2. Sensitivity analysis with the data excluding participants with AST>50 and ALT>50; AST>60 and ALT>60; or AST>70 and ALT>70

|  | Number of any type of cancer development  Adjusted hazard ratio  (95% confidence interval) | | | | | | | | | |
| --- | --- | --- | --- | --- | --- | --- | --- | --- | --- | --- |
|  | Men | | | | | Women | | | | |
|  | Very low  1^st^ quintile | Low  2^nd^ quintile | Middle  3^rd^ quintile | High  4^th^ quintile | Very high  5^th^ quintile | Very low  1^st^ quintile | Low  2^nd^ quintile | Middle  3^rd^ quintile | High  4^th^ quintile | Very high  5^th^ quintile |
| Retaining only participants with AST<50 and ALT<50 | | | | | | | | | | |
| All participants | 414 | 628 | 407 | 354 | 269 | 143 | 411 | 495 | 690 | 711 |
|  | 0.96 | 1.03 | Reference | 1.07 | 1.15 | 1.17 | 1.01 | Reference | 1.03 | 1.02 |
|  | (0.83–1.11) | (0.90–1.16) |  | (0.92–1.23) | (0.99–1.35) | (0.97–1.43) | (0.88–1.15) |  | (0.92–1.16) | (0.91–1.14) |
| Only abstainers | 126 | 153 | 92 | 64 | 32 | 87 | 229 | 257 | 356 | 314 |
|  | 0.93 | 0.95 | Reference | 0.83 | **0.65** | 1.19 | 0.97 | Reference | 1.04 | 0.99 |
|  | (0.70–1.25) | (0.73–1.23) |  | (0.60–1.15) | **(0.42–0.99)** | (0.92–1.54) | (0.81–1.17) |  | (0.88–1.22) | (0.84–1.17) |
| Only occasional drinkers | 57 | 79 | 61 | 45 | 30 | 28 | 86 | 94 | 120 | 129 |
|  | **0.63** | 0.72 | Reference | 0.71 | 1.05 | 1.04 | 1.11 | Reference | 1.04 | 1.04 |
|  | **(0.43–0.92)** | (0.51–1.00) |  | (0.48–1.05) | (0.67–1.65) | (0.67–1.61) | (0.82–1.50) |  | (0.79–1.36) | (0.79–1.36) |
| Only regular drinkers | 231 | 396 | 254 | 245 | 207 | 28 | 96 | 144 | 214 | 268 |
|  | 1.04 | 1.13 | Reference | **1.25** | **1.34** | 1.33 | 0.99 | Reference | 1.02 | 1.04 |
|  | (0.86–1.25) | (0.96–1.32) |  | **(1.05–1.50)** | **(1.11–1.62)** | (0.87–2.02) | (0.76–1.28) |  | (0.83–1.27) | (0.84–1.27) |
| Retaining only participants with AST<60 and ALT<60 | | | | | | | | | | |
| All participants | 467 | 631 | 408 | 356 | 271 | 152 | 411 | 495 | 691 | 711 |
|  | 0.96 | 1.03 | Reference | 1.07 | 1.15 | 1.15 | 1.00 | Reference | 1.03 | 1.02 |
|  | (0.83–1.10) | (0.90–1.16) |  | (0.93–1.23) | (0.99–1.35) | (0.95–1.40) | (0.88–1.15) |  | (0.91–1.15) | (0.90–1.14) |
| Only abstainers | 139 | 153 | 92 | 64 | 32 | 92 | 229 | 257 | 356 | 314 |
|  | 0.92 | 0.94 | Reference | 0.82 | **0.63** | 1.17 | 0.97 | Reference | 1.03 | 0.99 |
|  | (0.69–1.23) | (0.72–1.23) |  | (0.59–1.14) | **(0.42–0.97)** | (0.91–1.51) | (0.81–1.17) |  | (0.88–1.21) | (0.84–1.17) |
| Only occasional drinkers | 70 | 80 | 61 | 45 | 30 | 30 | 86 | 94 | 120 | 129 |
|  | **0.65** | 0.72 | Reference | 0.72 | 1.07 | 1.04 | 1.11 | Reference | 1.04 | 1.04 |
|  | **(0.45–0.94)** | (0.51–1.00) |  | (0.49–1.06) | (0.68–1.67) | (0.68–1.60) | (0.82–1.50) |  | (0.79–1.36) | (0.79–1.36) |
| Only regular drinkers | 258 | 398 | 255 | 247 | 209 | 30 | 96 | 144 | 215 | 268 |
|  | 1.03 | 1.13 | Reference | **1.26** | **1.34** | 1.28 | 0.98 | Reference | 1.03 | 1.04 |
|  | (0.86–1.24) | (0.96–1.32) |  | **(1.06–1.50)** | **(1.11–1.61)** | (0.85–1.93) | (0.75–1.27) |  | (0.83–1.27) | (0.84–1.27) |
| Retaining only participants with AST<70 and ALT<70 | | | | | | | | | | |
| All participants | 491 | 635 | 408 | 356 | 273 | 159 | 412 | 495 | 691 | 711 |
|  | 0.95 | 1.03 | Reference | 1.07 | 1.16 | 1.15 | 1.01 | Reference | 1.03 | 1.02 |
|  | (0.83–1.10) | (0.91–1.17) |  | (0.93–1.23) | (0.99–1.35) | (0.95–1.39) | (0.88–1.15) |  | (0.92–1.15) | (0.90–1.14) |
| Only abstainers | 146 | 153 | 92 | 64 | 32 | 95 | 230 | 257 | 356 | 314 |
|  | 0.91 | 0.94 | Reference | 0.82 | **0.63** | 1.14 | 0.98 | Reference | 1.03 | 0.99 |
|  | (0.68–1.21) | (0.72–1.22) |  | (0.59–1.14) | **(0.42–0.97)** | (0.89–1.47) | (0.82–1.17) |  | (0.88–1.21) | (0.84–1.17) |
| Only occasional drinkers | 75 | 80 | 61 | 45 | 30 | 31 | 86 | 94 | 120 | 129 |
|  | **0.66** | 0.71 | Reference | 0.72 | 1.06 | 1.03 | 1.12 | Reference | 1.04 | 1.03 |
|  | **(0.46–0.96)** | (0.51–1.00) |  | (0.49–1.06) | (0.67–1.66) | (0.67–1.58) | (0.83–1.51) |  | (0.79–1.36) | (0.79–1.35) |
| Only regular drinkers | 270 | 402 | 255 | 247 | 211 | 33 | 96 | 144 | 215 | 268 |
|  | 1.03 | 1.14 | Reference | **1.26** | **1.35** | 1.34 | 0.97 | Reference | 1.03 | 1.04 |
|  | (0.86–1.23) | (0.97–1.33) |  | **(1.05–1.50)** | **(1.12–1.62)** | (0.90–1.99) | (0.75–1.27) |  | (0.83–1.27) | (0.84–1.27) |

Models were adjusted for age; sex; body mass index; smoking status; alcohol consumption (only for all participants); exercise habits; medical histories of hypertension, diabetes, and fatty liver; family history of any type of cancer; and, the presence of hepatitis C virus (HCV) antibody, hepatitis B surface (HBs) antibody.

The numbers in bold represent that the p-value is less than 0.05.
